# *Brucella melitensis* B115-based ELISA to unravel false positive serologic reactions in bovine brucellosis: a field study

**DOI:** 10.1186/s12917-020-02278-7

**Published:** 2020-02-11

**Authors:** Adriana Trotta, Mariarosaria Marinaro, Margie Cirilli, Alessio Sposato, Rosanna Adone, Matteo Beverelli, Domenico Buonavoglia, Marialaura Corrente

**Affiliations:** 1 Department of Veterinary Medicine, University of Bari “Aldo Moro”, Str. Prov. per Casamassima Km 3, 70010 Valenzano, BA Italy; 2 Department of Infectious Diseases, Istituto Superiore di Sanità, Viale Regina Elena 299, 00161 Rome, Italy; 3 Istituto Zooprofilattico Sperimentale di Puglia e Basilicata (IZSPB), Sezione di Putignano, Contrada San Pietro Piturno, 70017 Putignano, BA Italy

**Keywords:** Brucellosis diagnosis, Serology, ELISA test

## Abstract

**Background:**

Brucellosis is a zoonosis whose incidence is not declining worldwide despite the global effort to control the disease. Accurate and precise diagnosis is a crucial step in any prophylaxis program but single tests to unequivocally detect animals infected with *Brucella* spp. are currently unavailable. In Italy, serological diagnosis of bovine brucellosis is performed with two official tests: a rapid agglutination test (i.e., Rose Bengal Plate test, RBPT) and a complement fixation test (CFT) that detect antibodies directed mainly to the smooth lipopolysaccharide (S-LPS). Neither of the two tests is able to avoid the detection of false positive serological reactions (FPSRs) caused by bacteria sharing S-LPS components with *Brucella* spp. and responsible for the single reactors (SR) phenomenon. A *B. melitensis* R strain-based ELISA showed a good diagnostic performance in unravelling FP animals; however, since a limited number of animals were analyzed in that study, a large field study was conducted here to discriminate between *Brucella*-infected from FP animals, with the final aim of reducing the unnecessary slaughter of the latter. An ELISA based on a R strain of *Brucella*, i.e., *Brucella melitensis* B115, was employed to measure specific IgG responses in a collection of bovine sera (*n* = 648). Sera were obtained from 180 farms (either officially brucellosis-free or not brucellosis-free) recruited during an extended period of time (2014–2018) and were preliminarily assayed with the official tests by the Italian Reference Centers and then subjected to the ELISA.

**Results:**

Negative sera, when subjected to the ELISA, gave O.D. values below the cutoff; SR sera, i.e. RBPT positive and CFT negative, as well as double positive (DP) sera, i.e. RBPT and CFT positive, gave O.D. values that were below the cutoff. All positive sera, i.e. from *Brucella*-infected animals, were RBPT positive and CFT positive (ICFTU ranging from 20 to 1280) and gave ELISA O.D. values above the cutoff.

**Conclusions:**

The *B. melitensis* B115-based ELISA systematically unravelled all false positive (FP) sera while confirming the diagnosis in *Brucella*-infected animals. Thus, the test employed in the present study may complement the official assays to avoid the costly slaughter of FP animals.

## Background

Brucellosis is an ancient and re-emerging zoonosis occurring worldwide [1]; it is caused by bacteria belonging to the genus *Brucella* which infect a variety of mammals and cause abortion and infertility in domestic animals [2]. Some *Brucella* species, such as *B. abortus* and *B. melitensis*, are mainly transmitted to humans by the consumption of contaminated dairy products and may cause a severe debilitating disease. Reduction of the global burden of human infection could be reached only by controlling animal disease [3, 4]. Control measures for animal brucellosis are different in different geographic areas and range from vaccination to test-and-slaughter programs although both approaches complement to reliable diagnosis [1]. Bacterial isolation and identification is clearly the gold standard diagnostic method but it is time consuming and impractical since it is performed on organs from slaughtered animals; in addition, the low isolation rate from infected tissues often results in false negatives [1, 5]. Serologic assays are rapid and simple systems to detect infected animals; several tests based on different principles have been developed worldwide to reach a good level of specificity and sensitivity although neither ideal nor unique serological test is available to precisely diagnose animal brucellosis [6–8]. Mediterranean countries are not brucellosis-free; in particular, in Italy the disease occurs in ruminants with low prevalence in Southern regions and, since vaccination is not allowed, the test-and-slaughter strategy together with sero-epidemiological surveillance programs are in force to control the disease in these areas. Diagnosis of bovine brucellosis is based on two official serological tests: a rapid agglutination test (i.e., Rose Bengal Plate test, RBPT) and a complement fixation test (CFT) [9]. Both tests are routinely performed by the Italian Reference Centers (Istituti Zooprofilattici, IZS) and use whole bacteria as antigen, i.e., *Brucella abortus* (S 99 strain), to detect antibodies directed against the immunodominant O-chain of smooth lipopolysaccharide (S-LPS) of *Brucella* [10]. The sequential use of both tests allows the detection of infected animals but false positive serological reactions (FPSRs) are also detected since other Gram-negative bacteria (e.g., *Salmonella spp*., *Escherichia coli* O157 or *Yersinia enterocolitica* O:9) share S-LPS components with *Brucella* spp. Indeed, *Y. enterocolitica* O:9 infections are frequent in bovine herds [11, 12] and generate FPSRs indistinguishable, by the official tests, from true positives [13]. The existence of FPSRs is a huge economical issue especially for brucellosis-free farms since brucellosis is a reportable disease and, according to the local Regulations operating in Apulia and Basilicata, the suspected presence of infected animals (RBPT positive/CFT positive or RBPT negative/CFT positive), but also the detection of potential FP animals, determines the loss of the brucellosis-free status and the slaughter of the seropositive animals. Cross-reactions have been reported not only in ruminants but also in pigs and humans because no individual specific test is available in any species [6, 11, 12, 14]. Thus, there is an urgent need for highly specific serological assays to implement brucellosis diagnosis and several tests based on other *Brucella* antigens have indeed been developed [15–17]. Rough (R) *Brucella* strains (such as *B.abortus* RB51, *B.melitensis* B115, *B.ovis* and *B.canis*), lacking the O-PS chain in the outer membrane of the cell wall, do not elicit cross-reactive antibodies against S-LPS and can be used to design more specific assays [18–21]. In particular, *B. melitensis* B115 was recently used as antigen to develop an ELISA [22] with good diagnostic performances but the paucity of bovine samples screened in that study prompted us to test it in a systematic field study. Thus, to specifically address this need, a large field study was conducted in a geographic area with low disease prevalence to unravel FP animals, with the final aim of reducing their unnecessary slaughter.

## Results

Serum samples were collected during the period 2014–2018, from 180 farms either officially brucellosis-free (163 farms) or not brucellosis-free (17 farms) and all located in two regions of South Italy, i.e., Apulia and Basilicata. Sera were first subjected by the IZS to the official assays, i.e. RBPT and CFT, were subdivided into four groups (A, B, C, D) and were then assayed by the ELISA (Tables 1 and 2). Negative sera (*n* = 259; group A, Table 1) were from officially brucellosis-free farms, they tested both RBPT and CFT negative and when subjected to the ELISA they all gave an O.D. value below the cutoff value (O.D. 0.143) which was previously determined by a ROC analysis [22] and was not significantly different from that calculated in the present study (O.D. 0.141; *p* > 0.05). A considerable number of SR, i.e. RBPT positive and CFT negative (*n* = 150; group B; Table 1), and of DP sera, i.e. RBPT and CFT positive (*n* = 134; group C; Table 1) were also tested and both groups gave O.D. values that were below the cutoff and not significantly different from those of group A (p > 0.05; Table 1). The CFT titers, expressed as International Complement Fixation Test Units (ICFTU), ranged from 20 to 80 in DP sera (n = 134; group C; Table 1). Indeed, post-mortem bacteriological and PCR analyses confirmed the absence of *Brucella* spp. in all samples tested (group B and C) while *Y. enterocolitica* O:9 was detected in 130 fecal samples from SR (n = 150; group B; Table 1) and in 100 DP animals (n = 134; group C; Table 1) (data not shown). Finally, Table 2 shows the comparison of all serological data obtained from *Brucella*-infected animals. All Group D sera (*n* = 105) tested RBPT positive and CFT positive (ICFTU ranging from 20 to 1280) and gave ELISA O.D. values above the cutoff. The individual ELISA O.D. values reported in Table 2 did not correlate with the relative ICFTU when subjected to linear regression analysis (data not shown).

**Table 1:** Comparison of the *B.melitensis* B115-based ELISA with Rose Bengal Plate Test (RBPT) and Complement Fixation Test (CFT) in brucellosis-free herds

ANIMALS	NUMBER	SEROLOGICAL TEST		
		RBPT	CFT	ELISA (O.D. ± SD)
Group A. Negative‡	259	Negative	Negative	0,063 ± 0,026
Group B. Single Reactors^^^	150	Positive	Negative	0,087 ± 0,013
Group C. Double Positive^§^	134	Positive	Positive°	0,086 ± 0,011

‡ animals tested negative to both official tests

^^^ animals tested positive only to one official test

^§^ animals tested positive to both official tests

° International Complement Fixation Test Units (ICFTU) ranged from 20 to 80

**Table 2:** Comparison of the *B. melitensis* B115-based ELISA with Rose Bengal Plate Test (RBPT) and Complement Fixation Test (CFT) in animals infected with *Brucella*

Group D^ animal number	RBPT	ICFTU°	ELISA O.D.
1	positive	20	0.300
2	positive	20	0.460
3	positive	20	0.279
4	positive	20	0.272
5	positive	20	0.268
6	positive	20	0.254
7	positive	20	0.268
8	positive	20	0.170
9	positive	20	0.175
10	positive	20	0.246
11	positive	20	0.246
12	positive	20	0.165
13	positive	40	0.288
14	positive	40	0.376
15	positive	40	0.220
16	positive	40	0.274
17	positive	40	0.318
18	positive	40	0.212
19	positive	40	0.154
20	positive	40	0.280
21	positive	40	0.240
22	positive	40	0.151
23	positive	40	0.244
24	positive	40	0.260
25	positive	40	0.385
26	positive	40	0.367
27	positive	40	0.302
28	positive	40	0.256
29	positive	40	0.280
30	positive	40	0.240
31	positive	40	0.300
32	positive	40	0.260
33	positive	80	0.280
34	positive	80	0.362
35	positive	80	0.300
36	positive	80	0.310
37	positive	80	0.206
38	positive	80	0.394
39	positive	80	0.226
40	positive	80	0.283
41	positive	80	0.310
42	positive	80	0.388
43	positive	80	0.396
44	positive	80	0.288
45	positive	80	0.304
46	positive	80	0.336
47	positive	80	0.340
48	positive	80	0.144
49	positive	80	0.144
50	positive	80	0.146
51	positive	80	0.161
52	positive	80	0.155
53	positive	80	0.153
54	positive	80	0.146
55	positive	160	0.370
56	positive	160	0.288
57	positive	160	0.296
58	positive	160	0.218
59	positive	160	0.146
60	positive	160	0.221
61	positive	160	0.240
62	positive	160	0.266
63	positive	160	0.260
64	positive	160	0.216
65	positive	160	0.392
66	positive	160	0.366
67	positive	160	0.210
68	positive	160	0.372
69	positive	160	0.396
70	positive	160	0.374
71	positive	160	0.292
72	positive	320	0.280
73	positive	320	0.294
74	positive	320	0.212
75	positive	320	0.280
76	positive	320	0.171
77	positive	320	0.237
78	positive	320	0.231
79	positive	320	0.299
80	positive	320	0.345
81	positive	320	0.320
82	positive	320	0.266
83	positive	320	0.392
84	positive	320	0.237
85	positive	320	0.374
86	positive	320	0.378
87	positive	320	0.244
88	positive	640	0.234
89	positive	640	0.232
90	positive	640	0.234
91	positive	640	0.389
92	positive	640	0.400
93	positive	640	0.385
94	positive	640	0.284
95	positive	640	0.378
96	positive	640	0.314
97	positive	640	0.300
98	positive	640	0.200
99	positive	1280	0.426
100	positive	1280	0.420
101	positive	1280	0.467
102	positive	1280	0.368
103	positive	1280	0.390
104	positive	1280	0.390
105	positive	1280	0.380

^Individual serum samples, n = 105, both RBPT and CFT positive, collected from 17 different *Brucella*-infected herds; the infection with *Brucella* spp. was confirmed by bacteriological and PCR analyses

° International Complement Fixation Test Units (ICFTU)

Finally, also the measurement of the percentile (99%) of the O.D. values obtained from brucellosis-free animals (*n* = 259; group A, Table 1) enabled to fully discriminate between uninfected and *Brucella*-infected animals (data not shown) [23].

The OIE international standard serum was included as positive control in the ELISA tests; it was tested in 10 replicates and gave always positive readings (O.D. 0.481 ± 0.005).

## Discussion

Despite the efforts made worldwide to control and eventually eradicate brucellosis, the disease remains one of the most common bacterial zoonoses with a constantly changing geographical distribution. Reducing the global burden of animal brucellosis will decrease the incidence of the disease in humans and compliance to control programs together with accurate diagnosis are instrumental to achieve this goal. The use of different strategies to control the disease, as well as the lack of diagnostic tests able to unequivocally diagnose the infection, impairs the effectiveness of control programs [1, 8]. In addition, the presence of FPSRs imposes the use of combined sero-epidemiologic methods and the development of better tests to implement diagnosis in animals. This is particularly urgent in areas with low disease prevalence such as the Mediterranean countries. Results reported here show that a *B. melitensis* B115-based ELISA is able not only to confirm the diagnosis made with the official tests but, most importantly, it can help unveil ambiguous FPSRs. According to the Italian prophylaxis Regulations, an animal is considered infected with *Brucella spp*. when testes both RPBT positive and CFT positive. However, in Southern Regions of Italy, that are not brucellosis-free, the local Regulations impose to evaluate both the anamnesis and the epidemiological data of farmed animals (especially those from officially brucellosis-free farms) that result SR or DP after completing the official serological tests. In fact, according to the local Regulations, if an animal testes RPBT positive and CFT negative (the so-called SR) both tests are repeated two weeks later [24] meanwhile the herd loses the official brucellosis-free status. Whether the second analysis confirms the first result (RPBT positive and CFT negative) or gives positive results for both tests, the animal is slaughtered even if the epidemiological evidences exclude the presence of *Brucella* infections in that herd. Cross-reactivity with other Gram-negative bacteria sharing the S-LPS antigens may explain the detection of SR or DP animals in those officially brucellosis-free herds that unexpectedly show seropositive animals during serological controls [25]. To complicate the issue, the herd whose brucellosis-free status has been lost, can reacquire it when all seropositive animals have been slaughtered and when none of the remaining animals tests positive to three consecutive serological tests: two made at a 3-weeks interval and a final one made 3–6 months later. Thus, the brucellosis-free status can be re-established not earlier than 5–7 months after notification (even in the presence of only one SR or one DP animal). For the considerable economic losses due to the slaughter of SR/DP animals and the lengthy suspension of the brucellosis-free status, the development of more specific serological tests, to precisely diagnose brucellosis, is highly desirable. Ancillary serologic tests to be performed alongside the official tests may serve the cause and the use of *Brucella* antigens other than the whole bacteria or S-LPS, has been exploited in the past [15, 16]. The *B. melitensis* R strain employed here proved to be a good antigen to unravel FP animals in a large field study conducted in a geographic area with low disease prevalence (prevalence of bovine brucellosis 2.06% in Apulia and 0.67% in Basilicata) [24].

The official serological assays performed by the IZS provide a dual level of information on specific antibody responses to *Brucella*: RBPT, which is used as a screening qualitative test, detects agglutinating antibodies while CFT provides a quantitative measure of complement fixing antibodies. In fact, sera with ICFTU equal to or above 20 are considered positive. In the present study the O.D. values measured with the ELISA did not correlate with the ICFTU, a finding consistent with previous observations [22, 26] and that is likely due to the different nature of the two tests. Indeed, the ELISA reported here likely measures the total amount of IgG directed against a plethora of *Brucella* antigens, within the bacterial extract. While this can be viewed as a limitation of the study, it suggests instead that this ELISA could be exploited in the future to dissect the whole humoral responses in infected animals by determining for instance: i) the level of antibody isotypes / subclasses specific to *Brucella* and ii) the antigens, other than the immunodominant S-LPS, against which those antibodies are produced during infection and disease.

## Conclusions

Brucellosis is a serious disease with implications for both international trade and public health [1, 4] and its incidence is not declining despite the effort made worldwide. It is also paradigmatic of zoonoses requiring a multidisciplinary and coordinated One Health approach to achieve the goal of eradication. Since new reservoir hosts in wildlife and new *Brucella* species are being discovered, it is a global responsibility to control the disease, at any level, to reduce chances for *Brucella* spp. to infect new hosts and to conquer new animal/environment/human interfaces [4]. Specific diagnosis is a crucial first step to unequivocally detect infected animals for their subsequent management. Until single reliable diagnostic tests become available, multiple tests based on different principles should be applied especially to sera giving discordant results. The ELISA employed in the present study may complement official tests when aspecific serological reactions occur during brucellosis testing, in order to avoid the costly slaughter of FP animals infected with other Gram-negative bacteria.

## Methods

### Preparation of B. melitensis B115 extracts

*B. melitensis* attenuated strain B115 was provided by the Veterinary Laboratories Agency (VLA) of Weybridge (U.K.) and cultured to prepare the bacterial extract according to previously described protocols [21, 22].

Briefly, the bacteria were cultured in 1 l of *Brucella broth* (Becton Dickinson, France) at 37 °C in aerobic conditions under stirring for 3 days. When the culture reached an optical density (OD) of 2.080, the broth was centrifuged at 5000 g (ALC PK131R centrifuge, Milan, Italy) for 20 min. The pellet was washed with saline solution, inactivated at 100 °C for 10 min, sonicated, centrifuged and the supernatant was dialysed against distilled water before measurement of the protein content as previously described by Corrente et al. 2015.

### Herds and serum samples

One hundred eighty herds from the South of Italy (Apulia and Basilicata regions) were recruited over an extended period of time, i.e. from 2014 to 2018. All animals were screened by the IZS during official brucellosis survey. A total of 648 sera were collected and subjected to the official diagnostic assays (RBPT and CFT) before testing them with the *B. melitensis* B115-based ELISA. The sera were subdivided into 4 different groups.

Group A: serum samples, both RBPT and CFT negative (*n* = 259), that were collected from 11 officially brucellosis-free herds;

Group B: serum samples from SR animals (*n* = 150) tested positive in RBPT and negative in CFT; they were collected from 102 different officially brucellosis-free herds. These animals were slaughtered according to the local Regulations and the IZS tested for the absence of *Brucella* spp. infection by using both bacteriological testing (PT/DIA/004) and real-time PCRs [9, 27]. In addition, fecal swabs were screened for *Yersinia enterocolitica* O:9 by official tests performed at the IZS (UNI EN ISO 10273:2017).

Group C: serum samples (*n* = 134), that tested positive in both serological conventional tests and were collected from 50 officially brucellosis-free herds; although epidemiological data indicated that they could be FP, these animals were slaughtered to fulfill the local Regulations. As for Group B, the absence of *Brucella* spp. infection was tested post-mortem and the screening for *Y. enterocolitica* O:9 was performed by IZS as previously described.

Group D: serum samples (*n* = 105) were both RBPT and CFT positive and were collected from 17 *Brucella*-infected herds. The animals were slaughtered and infection with *Brucella* spp. was confirmed by bacteriological and PCR analyses performed post-mortem by the IZS as described elsewhere [9, 27].

### Serological tests

Serological conventional tests were performed by the IZS according to international standard procedures [9] while the ELISA was carried out in the Laboratory of Bacteriology at the University of Bari according to a previously described protocol with some modifications [22]. Briefly, polysorp microtiter plates (Nunc, Milan, Italy) were coated with 100 μl of bacterial extract (25 μg of proteins/ ml) in carbonated buffer and incubated overnight at 4 °C under gentle shaking. The plates were then washed four times with PBS containing 0.05% Tween 20 (PBS-T) and wells were blocked for 150 min at 37 °C with 0.2% gelatin in carbonate buffer. After repeated washes, 100 μl of serum, diluted 1:100 in PBS-T, were added and the plates were incubated at 37 °C for 120 min. After washings, a rabbit anti-bovine antibody labeled with peroxidase (Sigma Aldrich, Milan, Italy) was diluted 1:3000 in PBS-T and added to the plates which were then incubated for 60 min at 37 °C. After final washings, an ABTS [2.2′-Azino-di-(3-ethylbenzothiazoline sulfonate)] solution (Sigma Aldrich, Milan, Italy) was added to each well and the plate was incubated at room temperature, without light for 20 min. The O.D. was measured at 405 nm using an automated ELISA reader. The negative samples (group A, *n* = 259) were used to determine the cut-off value of the ELISA test (i.e., the arithmetic mean of the O.D. of all negative samples plus 3 standard deviations).

The OIE international standard serum, supplied by the OIE Reference Laboratory for brucellosis at the VLA (Weybridge, UK), was used as positive control serum.

### Data analysis

The Microsoft Excel® 2010 program was employed to evaluate: i) the arithmetic mean and the standard deviation of O.D. values within a single group of animals; ii) the comparison of O.D. values between different animal groups (by chi square test with Yates correction); iii) the linear regression analysis between ICFTU and O.D. values in *Brucella*-infected animals (Group D); the percentile (99%) of O.D. values obtained in uninfected animals (Group A).

A *p* value below 0.05 was considered significant.

## Data Availability

The datasets used and/or analyzed during the current study are available from the corresponding author on reasonable request.

## Abbreviations

CFT: Complement Fixation Test
DP: Double positive
FP: False positive
FPSR: False positive serological reaction
ICFTU: International Complement Fixation Test Units
IZS: Italian Reference Centers (Istituti Zooprofilattici)
O.D.: Optical density
RBPT: Rose Bengal Plate test (rapid agglutination test)
S-LPS: O-chain of smooth lipopolysaccharide
SR: Single reactors
